# A trust-wide quality improvement programme to reduce out-of-area placements, length of stay and costs across inpatient mental health services

**DOI:** 10.1016/j.fhj.2025.100496

**Published:** 2025-12-16

**Authors:** Sarah McAllister, Marco Aurelio, Philip Baker, Lucy Brewer, Joanna Moore, Edwin Ndlovu, Angharad Rutley, Jamie Stafford, Amar Shah

**Affiliations:** East London NHS Foundation Trust, London, United Kingdom

**Keywords:** Quality improvement, Mental health, Inpatient, Flow

## Abstract

**Background:**

In the UK, rising demand for inpatient mental healthcare and fewer beds has led to persistently high occupancy, delayed admission and increased out-of-area placements. Consequently, only the most complex cases are admitted, contributing to longer lengths of stay.

**Methods:**

Between April 2024 and March 2025 East London NHS Foundation Trust ran a trust-wide, large-scale quality improvement programme across all geographical directorates, to eliminate out-of-area placements and reduce length of stay. The programme followed a structured improvement approach; directorates moved stepwise from identifying a quality issue through to implementing change ideas that resulted in improvement through testing.

**Results:**

Out-of-area placements reduced 93%, (from 66.5 to 4.6 per week), avoiding £8.5 million in costs. Average length of stay on London adult mental health wards fell by 17.5%, (53 to 43.9 days). Trust-wide bed occupancy decreased from 106% to 92%, and service users clinically ready for discharge reduced by 51%, (111 to 55 per week).

**Conclusion:**

Tailored interventions, real-time data, leadership and collaboration drove impact. Future work will focus on sustaining gains and enhancing community alternatives.

## Highlights

• Step-up/step-down beds, crisis house and robust triage linked to better flow outcomes.
• Bed routines and flow teams support faster transitions and system response.
• Locally tailored interventions enhance engagement and sustainability.
• Standardising core principles helped deliver consistent outcomes.
• Leadership, partnerships, data and daily management drove effective flow.

## Introduction

Demand for inpatient mental health services has grown in the last decade, with a 24% increase in people requiring hospital care from April 2016 to November 2023.^1^ Concurrently, inpatient mental health beds are at their lowest since 2011, with occupancy consistently above the Royal College of Psychiatrists’s (RCPsych’s) recommended 85%. Consequently, people may be provided care in inappropriate environments or face long waits in emergency departments. Increasingly, people needing inpatient psychiatric care are being placed in beds far from where they may usually live, in ‘out of area placements’ (OAP).^2^

Although OAP are appropriate in some cases, in many instances they are not and can result in poorer outcomes including longer length of stay, increased readmissions and higher rates of self-harm.^3^^,^^4^ Mental Health OAP also represent significant financial cost to the NHS, with £180 million being spent from April 2023 to March 2024, up from £102 million in the 12 months to March 2022.^2^

OAPs are a sign of system dysfunction, with bed pressures caused by care delivery issues such as too few acute beds, limited community care, staff shortages, long length of stay and wider determinants of health issues. Solving flow problems therefore requires a whole-systems approach.

The flow programme began in April 2024 in response to the over-reliance on OAP and increasing length of stay across the trust’s inpatient wards. The programme had two key aims:

• To reduce OAPs to 0 by October 2024.
• Reduce average length of stay (LoS) to 32 days by April 2025.

## Methods

This manuscript was prepared in accordance with the SQUIRE (Standards for Quality Improvement Reporting Excellence)^5^ 2.0 guidelines for reporting quality improvement work.

### Study context

East London NHS Foundation Trust (ELFT) provides mental health, community health, primary care, and specialist health services to a population of 2.2 million people across East London, and Bedfordshire and Luton. ELFT is structured into multiple directorates, each responsible for a geographical location or specialised service type.

In total, 40 adult inpatient mental health teams worked with systems partners from across the wider system. The characteristics of the wards are described below in Table 1.

**Table 1:** Characteristics of the wards.

Directorate	Ward type & number	Number of beds
Bedfordshire and Luton	One acute female ward	18
	Two acute male wards	44
	Two male PICUs	21
	One older adult ward	26
City and Hackney	Two acute female wards	38
	Two acute male wards	36
	One early intervention ward	14
Newham	Two acute female wards	34
	Two acute male wards	36
	Two triage wards (one male, one female)	30
Forensics	Two male medium secure wards	33
	Five male low secure wards	80
	Two male rehabilitation wards	33
	One male step-down ward	16
	One high dependency unit	11
	One female medium secure ward	15
Tower Hamlets	Two acute female wards	38
	Two acute male wards	38
	Two older adult wards	38

### Programme governance and learning system

Executive-level sponsorship for the programme was provided by the chief operating officer, deputy chief executive / director of integrated care and chief quality officer. At directorate level, the work was led by senior members of the directorate management team (DMT), including clinical and borough directors and borough lead nurses.

A monthly trust-wide learning session, chaired by executive sponsors, was held and brought directorates together to share learning and progress. Fortnightly local oversight spaces were also convened in each directorate, where local management teams reviewed learning from change ideas. Several corporate services supported the work, including finance, quality improvement, performance, people participation, data and analytics.

### Study design

**Fig. 1:**
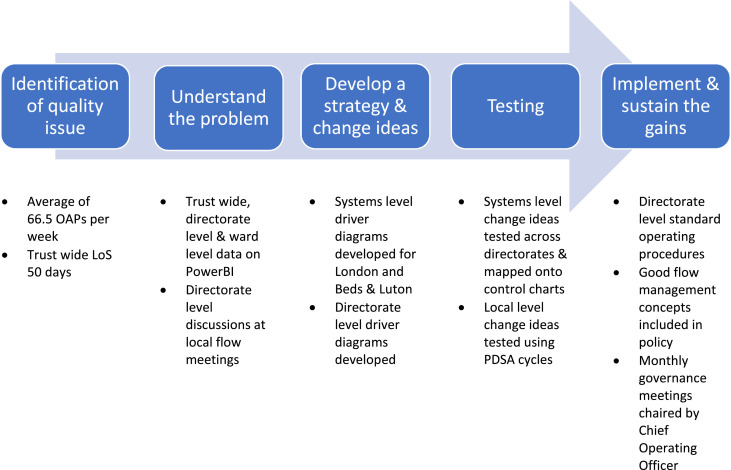
The sequence of improvement and steps taken at each phase.

This programme used quality improvement (QI) methods to tackle patient flow. QI has been used to tackle issues around flow in a range of healthcare settings.^6^^,^^7^ The programme followed a standard sequence of improvement, which has been widely used in other improvement work.^8^^,^^9^ This enabled the directorates to move stepwise from identifying a quality issue through to implementing change ideas that have resulted in improvement through testing (Fig. 1).

### Step 1: Identification of quality issue

The programme was initiated following recognition that OAPs and LoS were higher than desired; at 66.5 per week and 50 days respectively. Weekly spend on OAPs was an average of £370,937. Service users were also being cared for far from home, adversely impacting family and carer involvement and limiting the trust’s ability to maintain oversight and continuity of care.

### Step 2: Understanding the problem

A family of measures were chosen and a Microsoft PowerBi dashboard developed, which was accessible to all staff. Measures were displayed weekly over time on Statistical Process Control (SPC) charts, to understand patterns of variation and assess whether changes led to sustained improvements.^10^ The baseline for all measures ran from January 2024 to April 2024, when the work began, representing a sufficient baseline for SPC^11^ (Table 2).

**Table 2:** Programme level measurement plan.

Type of measure	Measure name	Operational definition
Outcome	Length of stay	Average LoS for discharges in a period. LoS is the count of days from start of admission to end of discharge. The mean is reported here to maintain consistency with NHS England conventions.^12^
Outcome	Out of area placements	Count of the number of service users in adult out of area mental health placements, excluding step-down and NHS provider beds per week.
Process	Bed occupancy	Number of beds occupied/total number of beds per week.
Process	Admissions	Count of the number of admissions per week.
Process	Discharges	Count of the number of discharges per week.
Process	Instances of clinically ready for discharge	Count of the number of service users who have a delayed discharge form completed on the electronic patient record system per week.
Balancing	Readmission within 28 days	Count of the number of service users re-admitted within 28 days of their initial admission.
Balancing	Trust-wide spend on OAPs	Total expenditure on out of area mental health placements across the trust.
Balancing	Trust-wide income from bed sales	Total income generated from selling bed capacity to other providers across the trust.

Data from SPC charts were triangulated with qualitative insights, which identified local barriers to discharge such as awaiting supported or emergency accommodation, telecare/adaptation, care coordination or public funding. This understanding enabled prioritisation of the key issues contributing to increased LoS and high use of OAPs.

### Step 3: Developing a strategy and change ideas

**Fig. 2:**
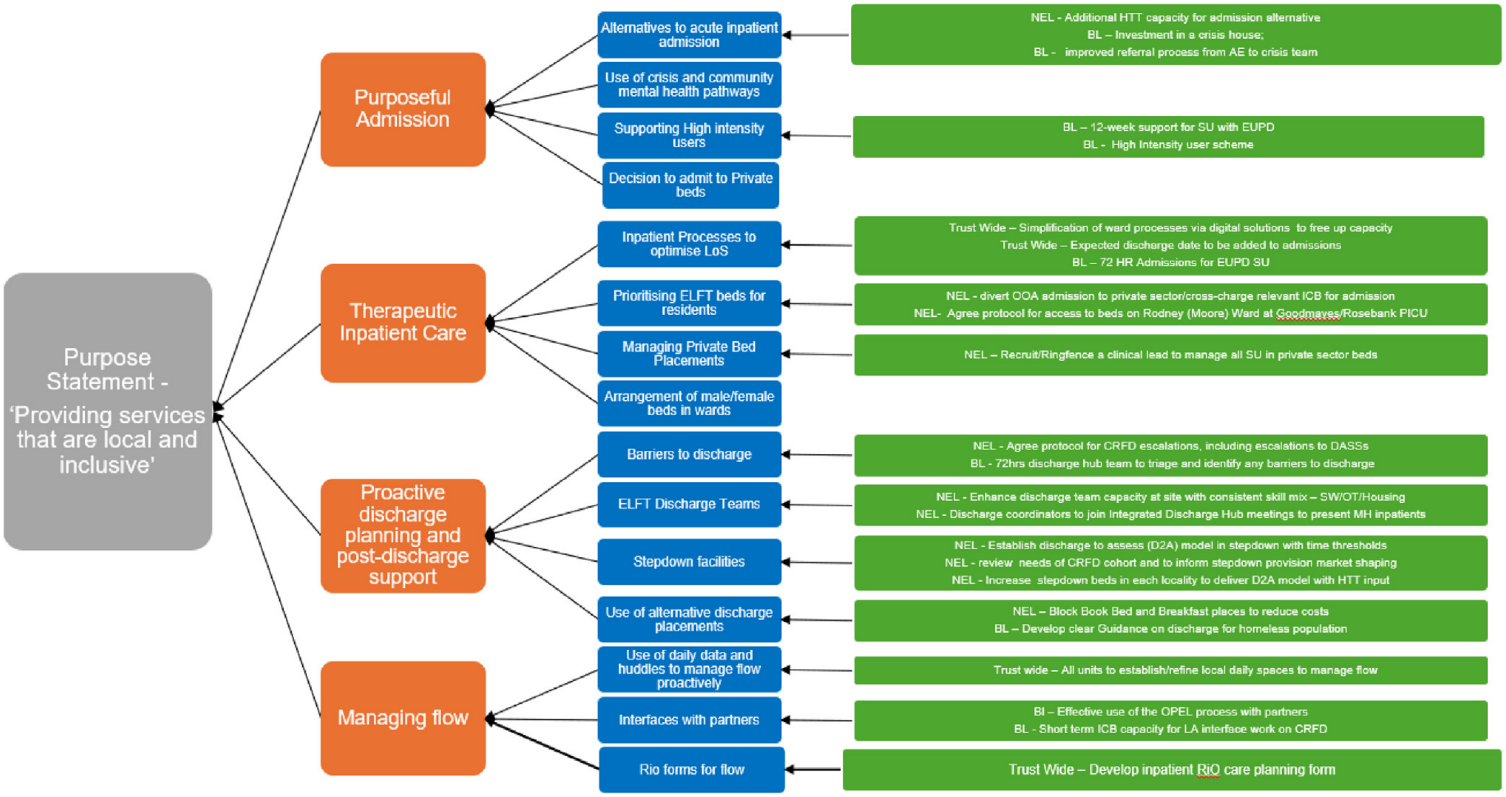
Programme-level driver diagram synthesised from the London and, Bedfordshire and Luton driver diagrams.

At programme level, a series of co-design workshops with clinical staff and services users were held to develop systems-level driver diagrams for both the London and Bedfordshire & Luton sites. Each directorate also developed its own local driver diagram, which aligned to system priorities but was tailored to suit each specific context and ensure local ownership over the work (Fig. 2).

### Step 4: Testing

Change ideas were tested using plan, do, study, act (PDSA) cycles; a four-stage, iterative testing method that involves planning the change, executing it, studying the results and acting on what is learned by either adopting, adapting or abandoning the change idea.^13^ Change ideas were tested across the whole inpatient pathway – preadmission, triage, admission and discharge, with those resulting in improvement presented in Table 3 below.

**Table 3:** Change ideas.

Location	Change ideas tested
Bedfordshire and Luton	Daily and weekly bed management meetings, decompression events, Home Treatment Team triages patients, discharge team with focused clinical support on OAPs, step-down beds, adding estimated discharge dates on all admissions.
City and Hackney	Daily, weekly and ad hoc bed management meetings, Home Treatment Team gatekeeps all admissions, discharge team with focused clinical support on OAPs, step-down and step-up beds.
Forensics	Reformatted weekly bed management meetings, clinically ready for discharge education, nursing admissions template, community nurses attend weekly ward rounds.
Newham	Daily and weekly bed management meetings, maintain the function of triage wards, step-down and step-up beds, discharge team with focused clinical support on OAPs.
Tower Hamlets	Red to Green huddles, weekly bed management meetings, Perfect Week events, Home Treatment Team gatekeeps all admissions, discharge to assess beds, step-up beds, crisis house, discharge team with focused clinical support on OAPs.

### Preadmission

#### Change idea 1: Step-up beds

To reduce hospital admissions, especially for those in acute mental health crises, most directorates (excluding Bedfordshire, Luton, and Forensics) introduced step-up beds. These provide short-term, intensive community-based care as an alternative to inpatient admission. Their goal is to stabilise individuals experiencing acute deterioration, delivering timely support without hospitalisation. The approach aims to lower acute admissions, ease system pressure, and ensure that inpatient beds remain available for service users with the most severe and immediate needs.

#### Change idea 2: Crisis house

In Tower Hamlets, this approach was extended via a crisis house, offering short-term, community-based accommodation and support for individuals experiencing a mental health crisis. The underlying theory was that access to a crisis house would reduce the need for inpatient admissions, support recovery in a more person-centred setting, and contribute to the number of inpatient beds available for those with the most acute need.

### Triage

#### Change idea 1: Maintain the function of triage wards

In the Newham Mental Health directorate, the triage ward model was significantly revised to ensure that all new admissions were assessed locally. The theory behind this idea was that it would prevent service users being admitted directly from the emergency department into a private sector bed, thus reducing the number of OAPs. It was also theorised that it would allow the directorate to determine the appropriate level of inpatient care and promote timely discharge by aligning care with planning an individual’s needs from the outset.

#### Change idea 2: Home Treatment Team gate keeps all admissions

In the remaining directorates, other than Forensics, the Home Treatment Team assumed the triage function, assessing referrals for admission and providing home-based care where appropriate. The theory behind this idea was that unnecessary inpatient admissions would be avoided, thus reducing the number of service users in inpatient beds and reducing the need for OAPs.

### Admission

#### Change idea 1: Estimated discharge date

Improving discharge planning was considered key, with Bedfordshire and Luton testing the practice of assigning an estimated discharge date (EDD) to all service users upon admission. The EDD served as a shared goal for those involved in delivering care helping to guide planning, identifying barriers to discharge early, and coordinate community support. The underlying theory was that establishing an EDD would promote proactive discharge planning, reduce LoS, and support more effective use of inpatient beds.

#### Change idea 2: Bed management and escalation meetings

Directorates introduced structured daily bed management discussions at ward level, enabling teams to identify and resolve discharge barriers. Unresolved issues were escalated to weekly directorate meetings, increasing visibility and accountability. In Tower Hamlets, this was formalised through Red to Green huddles,^14^ while others used existing safety huddles.^15^ Regular review and escalation aimed to promote timely discharge, reduce unnecessary occupancy and free up beds.

Weekly meetings were introduced to address complex cases, with senior leaders escalating systemic issues. In Bedfordshire and Luton and Tower Hamlets, initiatives like Perfect Week and Decompression events accelerated discharges and developed lasting improvements. The overall goal was to strengthen operational routines and cross-system coordination to improve discharge efficiency and expand local inpatient capacity.

### Discharge

#### Change idea 1: Discharge to assess and step-down beds

To address non-clinical discharge delays – such as pending social care or housing – most directorates, excluding Forensics, developed community-based alternatives for service users clinically ready to leave hospital. In Tower Hamlets, 12 discharge to assess beds were introduced, allowing patients to move out of acute care promptly, with assessments and care planning continuing at home or in community settings. This person-centred, recovery-oriented approach reduced unnecessary inpatient use.

Other directorates implemented step-down beds, offering short-term transitional support for individuals not yet able to return home, particularly those awaiting social care or accommodation. These initiatives aimed to reduce discharge delays, shorten length of stay, and ensure that inpatient beds were reserved for those with the most acute needs. The underlying goal was to enhance patient flow and efficiency by using community-based discharge pathways, ultimately reducing pressure on inpatient services and limiting out-of-area placements.

#### Overarching change idea: Dedicated flow and discharge teams across the inpatient pathway

A consistent theme across all directorates was the need for dedicated operational leadership to oversee flow along the entire inpatient pathway. In response, directorates tested dedicated flow and discharge teams responsible for providing real-time oversight, facilitating problem-solving, and supporting timely escalation of ELFT barriers to discharge. These teams monitored delays across both local ELFT inpatient beds and OAPs, ensuring that emerging issues were identified early and addressed proactively.

The underlying theory was that by embedding a consistent mechanism for oversight and accountability, flow and discharge teams could improve coordination, minimise delays, and ensure efficient use of local inpatient capacity. In doing so, they would support a reduction in LoS and reduce reliance on OAPs by enabling more timely transitions throughout the inpatient pathway.

#### Step 5: Implementation and quality control

To support implementation and quality control and prevent regression back to prior practices, several mechanisms were put into place. The change ideas shown to result in improvement were formalised into Standard Operating Procedures (SOPs) at directorate level. Improvement advisors supported directorates to complete these using a standardised template. This template drew on Roger’s Five Factors Framework – relative advantage, compatibility, complexity, trialability and observability.^16^

**Fig. 3:**
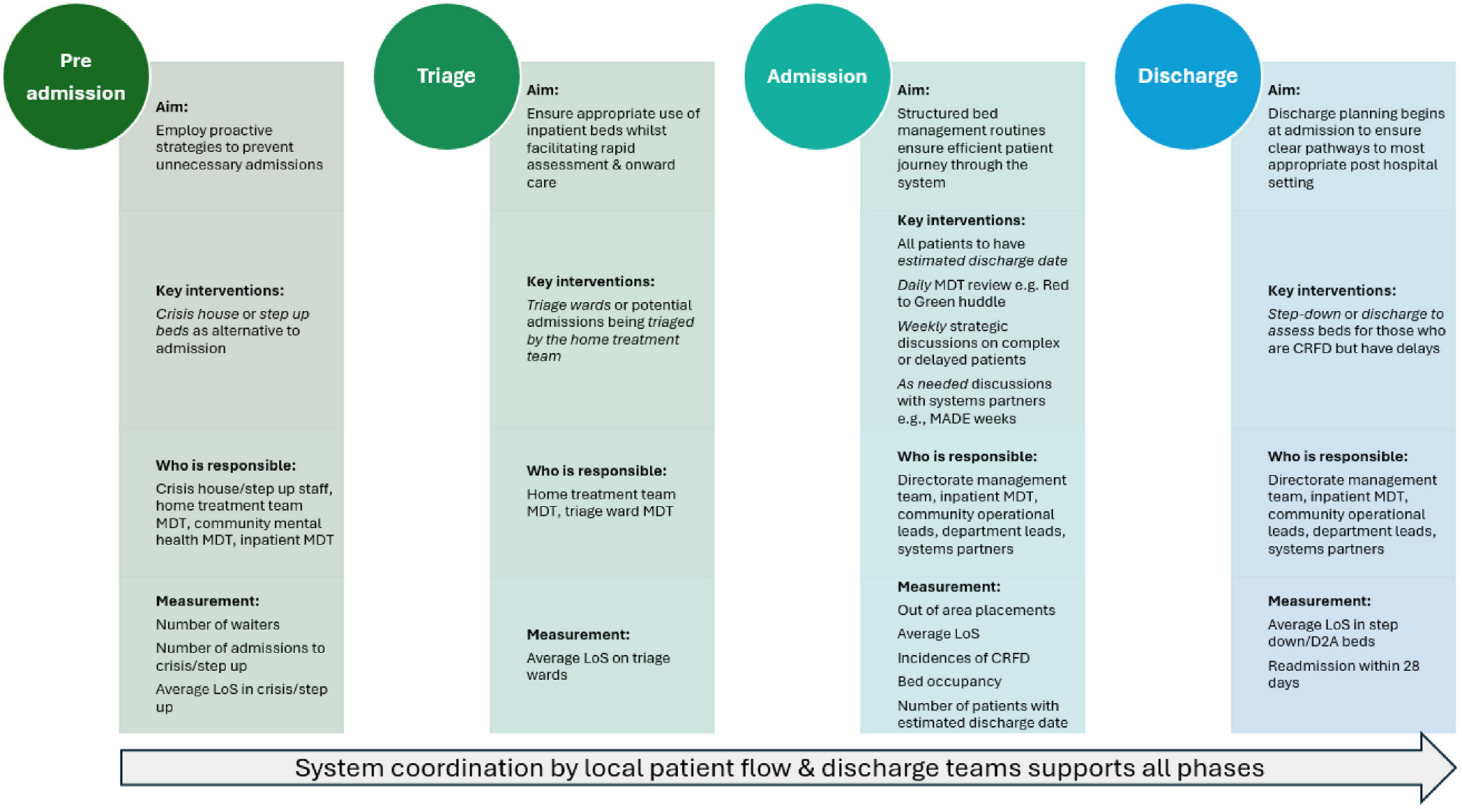
East London NHS Foundation Trust framework for flow management.

Drawing from this helped to ensure a standardised structure and prompted directorates to consider not just what the change was, but how and why it worked in their context. An example of the template can be found in Supplementary File 1. Local SOPs were held in directorates SOP SharePoint folders and shared at team away days, local clinical governance groups, directorate-wide communication emails and incorporated into staff inductions. At a trust-wide level, the change ideas shown to result in improvement were developed into a framework of flow management and incorporated into updated trust-wide inpatient policies (Fig. 3).

Additionally, ongoing governance structures were established. At directorate level, the work is reviewed at monthly directorate management meetings, ensuring that oversight remains active and responsive. Trust-wide oversight is maintained through continued review at the monthly operations meeting, chaired by the chief operating officer. This dual-level approach was intended to embed the improvements into routine practice and provide consistent strategic oversight. In addition, directorates regularly monitor key measures including OAPs and average LoS to detect early signs of deterioration and enable prompt intervention.

## Results

Improvements were seen in several measures across the programme.

### Out of area placements

Trust-wide OAPs, excluding the Forensic directorate who did not use OAPs, reduced by 93% from an average of 66.5 each week to 4.6. Across the wards in London, OAPs were reduced to zero. In Bedfordshire and Luton, there was a 74% reduction from 21 a week to five. Estimated spend on OAP reduced by 93% from an average of £370,937 to £28,242 each week, resulting in £8.5 million in cost avoidance in 2024/2025.

### Length of stay

Average LoS did not reduce trust wide. Across adult mental health wards in London, there was a 17.5% reduction from 53 days to 43.9 days. Two units (Newham and Forensics) and nine wards also saw individual reductions in LoS.

Newham saw a 25% reduction directorate wide, from 41 to 30 days. Across their acute wards there was a 28.5% reduction from 68 to 49 days, with triage wards seeing a 44% reduction to 9 days. Two acute wards in Bedfordshire and Luton saw a 53% and 57% reduction in average LoS, from 49.4 days to 23.3 days and 54 days to 23 days. In City and Hackney, two acute admission wards saw reductions in average LoS with decreases of 60% and 61%, respectively. On the first ward, the average LoS reduced from 74 days to 29 days, while on the second ward it decreased from 63 days to 24.5 days. Another two wards in Tower Hamlets also saw reductions. One older adult ward reduced LoS by 39%, from 118 days to 67 days and an acute admissions ward reduced LoS by 46%, from 49 days to 22.5 days.

Although not included in the aggregated outcome measure due to the distinct context of forensic services, the average LoS on discharge across the trust’s forensic units reduced by 45%, from 1,806 days to 966 days. Forensic mental health wards provide care for individuals with complex mental health needs who are also involved with the criminal justice system; with much longer LoS than the general population (Table 4 presents a summary of the results).

**Table 4:** Summary of results.

Measure	Baseline	End of testing	Change
Number of out of area placements	Average of 66.5 per week	Average of 4.6 per week	93% reduction across trust
Average length of stay across ELFT	Average of 50.6 days	Average of 50.6 days	No change
Average length of stay across London	Average of 53 days	Average of 43.9 days	17% reduction across all London adult mental health units
Average length of stay across Forensics	Average of 1,806 days	Average of 966 days	45% reduction across the Forensics unit
Percent of occupied beds	Average of 105% per week	Average of 93.2% per week	11.2% reduction across London, and Bedfordshire and Luton
Admissions	Average of 78.2 per week	Average of 68 per week	13% reduction across London, and Bedfordshire and Luton
Discharges	Average of 76.4 per week	Average of 74.3 per week	2.7% reduction across London, and Bedfordshire and Luton
Instances of clinically ready for discharge	Average of 111.9 per week	Average of 55.1 per week	50.7% reduction across London, and Bedfordshire and Luton
Readmission within 28 days	Average of three per week	Three per week	No change

**Fig. 4:**
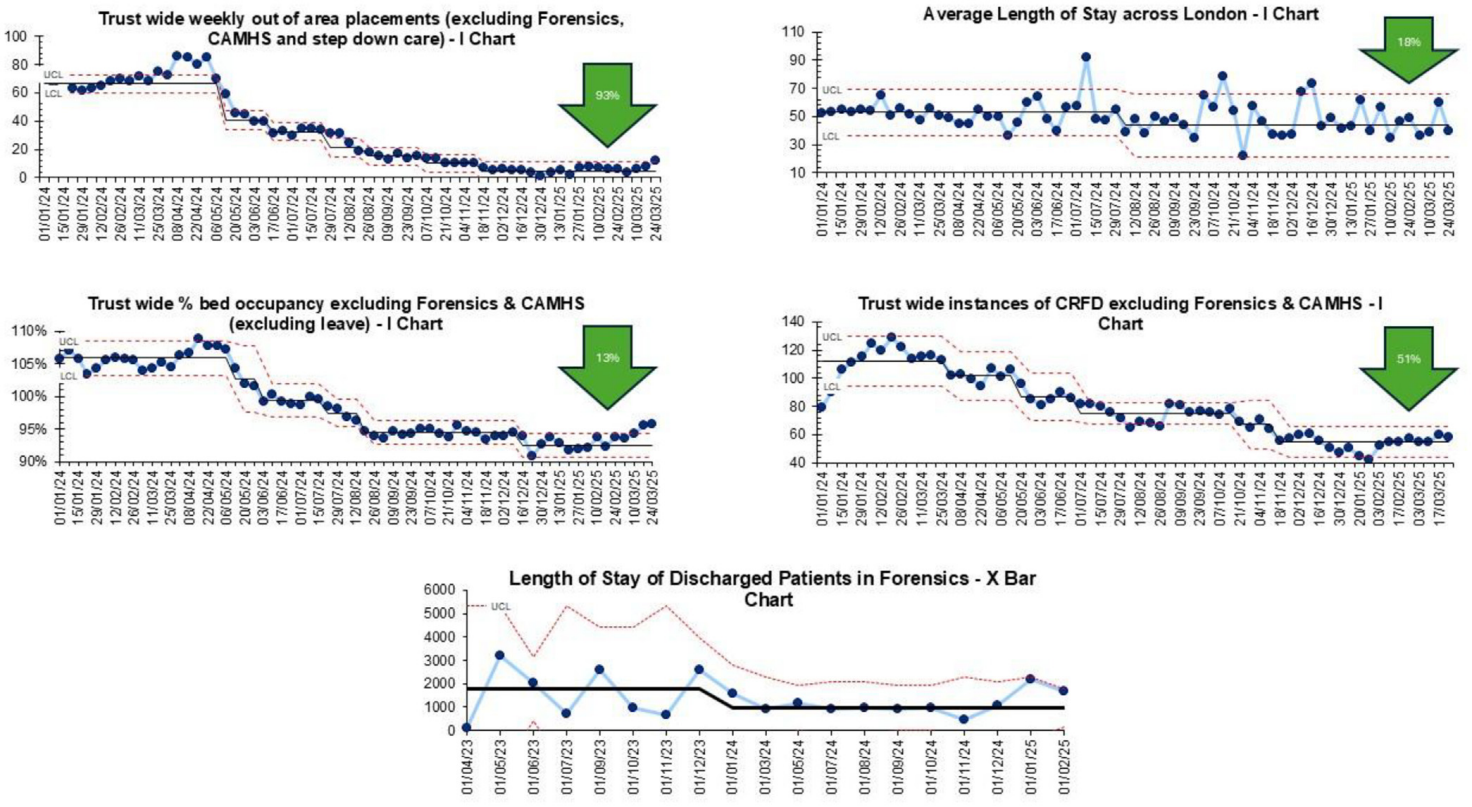
Dashboard of measures showing programme improvements.

Trust-wide bed occupancy reduced from 106% to 92%. It is noted that this remains above the recommended RCPysch guidance on safe levels of occupancy, which is 85%. Trust-wide instances of service users clinically ready for discharge reduced by 51% from 111 to 55 each week. Trust-wide admissions reduced from 78.2 to 68 per week and trust-wide discharges reduced from 76.4 to 74.3 per week. SPC analysis confirmed that reductions in length of stay reflected special cause signals, independent of the small changes in admission and discharge rates. Trust-wide readmissions within 28 days remained stable at an average of three per week (Fig. 4).

## Discussion

This paper outlines a large-scale QI programme aimed at eliminating OAPs across ELFT and reducing LoS to 32 days. The programme used a systematic approach, following the sequence of improvement to develop and test change ideas supporting patient flow across the inpatient pathway. This approach aligns with established QI frameworks^13^ and large-scale organisational management principles that promote sustained improvements.^17^

The key change ideas linked to improvements included step-up and step-down beds, a crisis house, structured triaging and integration of estimated discharge dates from admission. Operational improvements, such as revised bed management routines and dedicated flow and discharge teams, also played a critical role in supporting timely transitions and improving overall system responsiveness. These findings align with a growing evidence base demonstrating the benefits of alternative models of mental healthcare. In the UK, women’s crisis houses have been shown to provide comparable outcomes to hospital admissions at similar cost, with enhanced patient satisfaction when care aligns with patient preference. While service user satisfaction data were not collected as part of this programme – representing a notable limitation – the existing literature suggests that patient-centred alternatives can deliver both clinical effectiveness and improved user experience when designed with service user needs in mind.^18^

Similarly, mental health triage systems have demonstrated benefits in reducing inpatient length of stay, improving emergency department flow, and increasing consistency in clinical decision making.^19^ The integration of estimated discharge dates alongside bed management protocols can improve discharge planning and patient flow by fostering multidisciplinary coordination. However, the effectiveness of these approaches relies on accurate discharge forecasting and sustained team engagement; failure in these areas can lead to delayed discharges and increased bed pressures.^20^^,^^21^ Therefore, while our programme aligns with broader evidence supporting these models, continued focus on fidelity and staff collaboration remains crucial to maintaining and extending improvements.

Balancing fidelity with local adaptation is a recognised challenge in implementing change at scale.^22^ While local tailoring may enhance engagement and sustainability, standardisation of core practices is essential for delivering consistent outcomes across complex systems.^23^ To address this tension, a flow framework was developed (as previously shown in Fig. 3) aligned to the key phases of the inpatient pathway – preadmission, triage, admission and discharge – and incorporated into policy. The framework specified the essential change concepts and defined responsibilities at each stage, supporting proactive patient flow, timely triage, structured inpatient care, and early discharge planning. Approval by a multidisciplinary consultation group ensured the framework reflected operational realities and gained strategic ownership.

Despite focused efforts to reduce LoS across the organisation, trust-wide improvements have not yet been realised. Variability in local testing and implementation, systemic barriers and contextual factors often hinder uniformed progress in improvement work.^24^ Within ELFT, some directorates have encountered greater challenges in reducing both LoS and OAPs, which reflects differences in local service configuration such as access to step-down or step-up beds and systemic challenges like varying access to social care and housing stock. This type of variation is well documented, where progress relies not only on specific interventions, but also the local system’s readiness for change.^25^ In response, we are continuing work across the trust to focus on reducing LoS outside the remit of this programme.

The flow framework was underpinned by four key principles that were identified through discussions with the senior leadership of each directorate. These principles include leadership commitment, collaborative system working, daily management and adaptability, and data-driven decision-making. These principles reflect core components of effective flow management as described in the improvement literature^26^ and were instrumental in guiding both the strategic and operational aspects of the work.

Leadership commitment emerged as a core driver for change, with senior leaders such as the clinical and borough directors playing a visible and active role in articulating the case for change and being readily available on the frontline to support clinicians in flow management in the early stages of the work. Leadership engagement has been widely recognised as a critical success factor in quality improvement initiatives.^8^ Studies have shown that leadership behaviours, such as modelling improvement principles and creating psychological safety for teams, are associated with greater adoption of change and sustainability of improvements.^9^^,^^27^

Despite this, we found that having sustained involvement of senior leaders in day-to-day operational flow management was unsustainable. This aligns with findings from the improvement literature, which suggest that while visible leadership can accelerate early adoption, long-term sustainability depends on embedding robust routines, accountability structures, and empowering unit-level management and frontline teams to lead daily operations.^28^ Establishing standard work processes, clearly defined communication channels and mechanisms for continuous feedback, such as the daily and weekly meeting structures, were essential in maintaining momentum as senior leaders transitioned into a strategic oversight role. This aligns with literature that suggests creating interconnected routines that engage all levels of staff are essential to improving patient flow.^29^

Collaboration with system partners, including community teams, local authorities, social care and housing, was helpful in ensuring that patient care was aligned across the whole system. Evidence suggests that integrated care systems and inter-organisational collaboration are essential for managing flow effectively, especially for patients with complex needs.^26^ Nevertheless, cross-organisational working requires high levels of trust, shared goals and consistent communication, all of which can be challenging to sustain across organisational boundaries.^28^ This was evident in some directorates, where there was variation in the strength of relationships with system partners. Events such as Perfect Week and Decompression events provided opportunities to build and cement these relationships, while a QI approach enabled directorates and partners to test and learn together in a structured yet flexible way.

Importantly, the QI approach supported collaborative working not by prescribing solutions, but by creating space for joint problem-solving. As Johnson and colleagues^29^ note, improving flow is inherently relational; trust and collaboration are essential and, without them, technical solutions are unlikely to succeed. Efforts to direct change through top-down methods are unlikely to succeed in the long term. Instead, staff and partners need to learn their way into new ways of working through dialogue, experimentation and reflection. The use of QI methods supported this social learning process by enabling teams to make sense of local challenges, build ownership of change, and develop contextually appropriate solutions through iterative testing.

This paper shows how QI methods can enhance clinical outcomes while improving resource use and cost efficiency. Traditionally focused on safety and experience,^15^ QI is now recognised for its role in financial sustainability.^30^ Through this programme, weekly spending on private OAPs reduced by 93%, saving £8.5 million. This demonstrates QI’s potential to advance the healthcare triple bottom line – optimising outcomes and delivering environmental, social, and financial value – by improving local care, reducing strain on services, and promoting sustainable, high-value healthcare.^31^ Aligning QI efforts with this broader value framework supports the shift towards more sustainable healthcare.

## Limitations

This programme has limitations that should be considered alongside its findings. Firstly, although reductions in OAPs and average LoS across London directorates were observed, the testing of multiple interventions in parallel makes it difficult to attribute improvements to specific change ideas or PDSA cycles. This reflects a known limitation in evaluating complex interventions, where it can become difficult to attribute improvement to any specific component when multiple components are implemented simultaneously.^32^ More broadly, this also highlights a limitation of QI methodologies in that they are well suited to identifying correlations between interventions and outcomes, but are not designed to establish causation.^33^ Future research could seek to isolate the effects of specific interventions by employing a more structured evaluative design. This could include using Planned Experimentation,^34^ which could help to identify the components or combinations of interventions that were most influential.

While quantitative data on bed use, cost and LoS were analysed, no formal evaluation of staff or service user experience was conducted. Anecdotal feedback suggested improved continuity of care and reduced disruption for those treated closer to home, yet these outcomes need systematic qualitative study. Evidence from other research indicates that similar interventions – crisis houses, structured triage, and estimated discharge dates – can improve staff wellbeing and patient satisfaction by reducing delays.^18^^,^^35^^,^^36^ These findings highlight the potential for such models to enhance experience and engagement, though further evaluation is required in this programme’s context.

## Conclusion

This large-scale QI programme demonstrated that a system-wide QI approach can significantly reduce OAPs and LoS. Between April 2024 and March 2025, weekly use of private OAPs reduced by 93%, avoiding £8.5 million in cost. Reduced reliance on OAPs also contributed to improved equity of access, enabling service users, including those with complex needs, to receive care closer to home. By testing change ideas across the inpatient pathway, the programme addressed operational and clinical barriers while maintaining focus on patient-centred care.

Key to the programme’s success was the use of targeted, contextually relevant interventions such as step-up and step-down beds, revised triage models, structured bed management routines and strengthened oversight by flow and discharge teams. These interventions were tested locally, enabling directorates to adapt the core concepts to their specific needs. The integration of leadership modelling desired behaviours, real-time data monitoring, daily flow discussions and collaborative engagement with systems partners further supported and sustained improvement. The programme highlights the importance of having a cohesive, trust-wide framework for flow management that allows for local adaptation while maintaining strategic alignment.

Future work will focus on sustaining these gains and further working to reduce LoS across all inpatient wards. In parallel, continued work in community-based alternatives to care and cross-sector collaboration will be essential to ensure that reductions in inpatient use are safe, effective and person centred.

## Data availability statement

The data that support the findings of this study are available from the corresponding author upon reasonable request.

## Ethics approval and consent to participate

This project was conducted as a quality improvement (QI) initiative aimed at improving care within our local services. It did not involve any interventions outside standard clinical practice, did not pose additional risk to patients, and used routinely collected data. As such, it was not considered research and did not require formal ethics approval or individual consent in line with Health Research Authority (HRA) guidance.

## Funding

This research did not receive any specific grant from funding agencies in the public, commercial or not-for-profit sectors.

## CRediT authorship contribution statement

**Sarah McAllister:** Writing – original draft, Project administration, Methodology, Investigation, Formal analysis, Data curation, Conceptualization. **Marco Aurelio:** Writing – review & editing, Writing – original draft, Supervision, Methodology, Conceptualization. **Philip Baker:** Writing – review & editing, Validation. **Lucy Brewer:** Writing – review & editing, Validation, Investigation. **Joanna Moore:** Writing – review & editing, Validation. **Edwin Ndlovu:** Writing – review & editing, Validation. **Angharad Rutley:** Writing – review & editing, Validation, Investigation. **Jamie Stafford:** Writing – review & editing, Methodology, Investigation, Conceptualization. **Amar Shah:** Writing – review & editing, Supervision, Methodology, Conceptualization.

## Declaration of competing interest

The authors declare that they have no known competing financial interests or personal relationships that could have appeared to influence the work reported in this paper.

## Appendix Supplementary materials

Image, application 1
