# Supplementary material for: A trust-wide quality improvement programme to reduce out-of-area placements, length of stay and costs across inpatient mental health services
Source: Future Healthc J. 2025 Dec 16;13(1):100496. doi: 10.1016/j.fhj.2025.100496 (PMC12887397; doi:10.1016/j.fhj.2025.100496)
Supplement: Supplementary file 1 [file mmc1.docx]

| **Change idea** | | | |
| --- | --- | --- | --- |
| **Please describe the change idea** |  | | |
| **Why did the idea work?** |  | | |
| **Where was the idea tested e.g., ward, community, other** | | | |
|  | | | |
| **What are the steps involved in this work?** | | | |
| **What** | | **Who** | **When** |
|  | |  |  |
| **How will you know the idea is still working (measurement)** | | | |
|  | | | |
| **Changes to infrastructure (environment, policies, way people work, knowledge, skills)** | | | |
|  | | | |
| **What benefits did you see from this idea?** | | | |
|  | | | |
| **Challenges needed to be overcome?** | | | |
|  | | | |

*If appropriate, create a flow chart that clearly maps out the steps of the process
